# Case of Poisoning from the Seed of the Diospyros Virginiana

**Published:** 1840-03-28

**Authors:** William Zollickoffer


*Case of poisoning from the seed of the Diospyros Virginiana.* By William Zollickoffer, M. D.

*Middleburg*, (*Md.*) *March* 21, 1840.

To the Editors of the Medical Examiner.

*Gentlemen*:—The early part of December, 1838, Mr. Koouts sent for me to see his little son, who, on my arrival, I found complaining of great pain in the epigastric and abdominal regions. Before I saw him, in the act of vomiting, he had thrown up fifty-five seeds of the diospyros virginiana, which you know are very large. I administered an emetic of the sulphate of zinc, which effected the disgorgement of but fifteen more. Supposing, from this circumstance, in conjunction with there being an exceedingly hard knot in the region of the umbilicus, of considerable size, that they had passed into the bowels, I directed the free use of castor oil, until copious dejections were produced, by which, in three days, I effected the dislodgement of four hundred and fifty-two seeds more. As the seeds passed off, the hardened tumour gradually lessened, and at the end of the third day entirely disappeared. For several days after, a seed or two was occasionally discharged. The child's digestive organs seemed rather impaired, no doubt, from the gastro-intestinal debility produced by the distention, effected by so large a quantity of seed, of the usual size of the virginiana, being partially impeded in the intestinal canal for about four days from the time they had been swallowed. In order to restore his appetite, and impart tone to his stomach and bowels, I directed the use of Huxham's tincture three times a day, and the occasional use of a dose of castor oil. The child has since enjoyed good health.

I have given you the particulars of the above case, merely to show how large a quantity of foreign matter can be retained in the bowels without being productive of serious consequences, as sometimes happens from a very slight cause. You can use it as you please.

